# Prebiotic potential of green banana flour: impact on gut microbiota modulation and microbial metabolic activity in a murine model

**DOI:** 10.3389/fnut.2023.1249358

**Published:** 2023-10-31

**Authors:** Ga Hyeon Baek, Yu-Jeong Kim, Yukyung Lee, Suk-Chae Jung, Hwi Won Seo, Jun-Seob Kim

**Affiliations:** ^1^Department of Nano-Bioengineering, Incheon National University, Incheon, Republic of Korea; ^2^Infectious Disease Research Center, KRIBB, Daejeon, Republic of Korea; ^3^Biosystems and Bioengineering Program, University of Science and Technology (UST), Daejeon, Republic of Korea; ^4^B2S Company Co., Ltd., Seoul, Republic of Korea; ^5^Department of Bioengineering, University of Illinois Urbana-Champaign, Urbana, IL, United States

**Keywords:** green banana flour, prebiotics, beneficial bacteria, gut microbiota community, metabolic pathway prediction

## Abstract

**Introduction:**

Green banana flour can be used as a prebiotic due to its ability to promote gut health and provide several health benefits. In this study, we investigated whether feeding mice green banana flour at different doses would alter intestinal microbiota composition.

**Methods:**

We fed C57BL/6N mice either a Low-dose (500 mg/kg/day) or High-dose (2000 mg/kg/day) of green banana flour daily for 3 weeks, and fecal samples were collected on days 0, 14, and 21 for microbiota analysis.

**Results:**

Our results showed that the composition of intestinal microbiota was significantly altered by day 21, regardless of the dose. Notably, the consumption of green banana flour increased the presence of beneficial bacteria, including Coriobacteriaceae_UCG-002, Turicibacter, Parasutterella, Gastranaerophilales_ge, and RF39_ge. These changes in the intestinal microorganisms were accompanied by increased biological processes such as amino acid biosynthesis and secondary metabolite biosynthesis. Conversely, the consumption of green banana flour resulted in a decrease in biological processes related to carbohydrate degradation, glycerol degradation, and similar functions.

**Discussion:**

These results emphasize the potential of green banana flour as a prebiotic that can benefit the gut microbiome.

## Introduction

Trillions of microorganisms coexist within the human body, comprising a diverse ecosystem of beneficial and harmful bacteria. These microorganisms play a pivotal role in human health and disease by actively participating in metabolic processes, pathogen defense, immune system regulation, and influencing various physiological functions (1). The advent of next-generation sequencing (NGS) methods has provided valuable insights into the abundance, diversity, and functional significance of the human microbiome (2). Since the late 2000s, extensive research has highlighted the microbiome’s involvement in crucial physiological processes, including nutrient absorption, drug metabolism regulation, immune system modulation, brain and behavioral development, and prevention of infectious diseases (3–5).

In recent years, the significance of intestinal health in boosting immunity and promoting skin health has sparked interest, leading to the emergence of various biotic products such as probiotics, prebiotics, synbiotics, and postbiotics. Prebiotics are non-digestible carbohydrates that serve as a substrate for the gut microbiota, demonstrating resistance to gastric acid and digestive enzymes, thus arriving intact in the intestine. Here, they undergo bacterial fermentation, encouraging the proliferation of beneficial microflora, and providing substantial health advantages (6–8). They are prevalent in an array of dietary sources including diverse fruits, vegetables, cereals, and legumes such as tomatoes, bananas, various berries, sweet potatoes, asparagus, allium vegetables, chicory, leafy green vegetables, pulses, oats, flaxseed, barley, and wheat (8).

Among these, bananas are one of the most extensively cultivated tropical fruits worldwide, with over 1,000 varieties produced. However, a significant portion of the banana harvest goes to waste due to consumers’ prevalent preference for ripe bananas. Fresh green bananas contribute substantially to the overall loss and are generally unpalatable due to the presence of soluble phenolic compounds, such as tannins, imparting an astringent taste (9). Consequently, researchers have explored alternative derived products from green bananas, such as green banana flour and green banana biomass, to mitigate waste. Green banana flour, in particular, has garnered attention within the food industry due to its high resistant starch (RS) content, ranging up to 68% w/w (10). Upon reaching the colon, RS undergoes fermentation by the intestinal microbiota, yielding short-chain fatty acids (SCFAs) such as acetate, propionate, and butyrate (11). RS exhibits diverse therapeutic and preventive effects, including colon cancer prevention, blood glucose reduction, cholesterol reduction, fat accumulation inhibition, gallstone formation reduction, and enhanced mineral absorption (12).

Despite previous studies discussing the prebiotic effects of green banana flour, which is rich in RS, limited research has been conducted to substantiate significant changes in the gut microbiome and perform comprehensive metabolic pathway analyses through *in vivo* studies (10). To validate the effectiveness of green banana flour as a prebiotic, further investigations are warranted to elucidate its precise impact on the gut microbiome. This study aims to investigate the effects of green banana flour consumption on mice, with a specific focus on validating alterations in the composition of the intestinal microbiota and identifying associated metabolic pathways that may hold relevance to specific diseases. Our findings aim to provide compelling evidence of the prebiotic potential of green banana flour. Moreover, our research demonstrates that green banana flour can be applied as a prebiotic or functional food ingredient through the improvement of gut microbiota, and it provides a solution to the environmental issues caused by the disposal of unused green bananas, thereby emphasizing its novel contribution to the field of environmental science.

## Materials and methods

### Animal and experimental design

The experiments were conducted under the approved protocols by the Institute Animal Care and Use Committee of Korea Research Institute of Bioscience and Biotechnology (AEC-22255). After a quarantine period, the animals were acclimated for 7 days before being isolated into groups. Throughout the study, the mice were housed in a room with controlled environmental conditions, including a temperature range of 20.5°C–23.2°C, humidity range of 36.2%–56.3%, and a 12 h dark/light cycle. The animals were housed in stainless steel mesh cages with wood embedding, accommodating two to three mice per cage. They had unrestricted access to rodent chow (2018S; Envigo, Madison, WI, United States) and filtered water. The diet fed to mice contained 3.1 cal/g and consisted of 44.2% carbohydrates, 18.4% protein, 6.0% fat, and 3.8% fiber (Supplementary Table S1).

Fifteen 8 weeks-old male C57BL/6N mice (ORIENT Bio, Suwon, Republic of Korea) were purchased and divided into three groups (*n* = 5/group): (1) control group, where the control animals were administered distilled water as a vehicle; (2) low-dose group, where the mice were treated with 500 mg/kg body weight of green banana flour dissolved in suspended water; (3) high-dose group, where the mice were treated with 2,000 mg/kg body weight of green banana flour suspended in distilled water. The doses of 500 and 2,000 mg/kg body weight correspond to approximately 42 and 166 mg/kg, respectively, when calculated based on FDA guidance as equivalents to the human dose (13). The substances were administered orally via gavage, in a volume of 200 μL, for a duration of 21 days. The nutritional information of green banana flour is listed in Supplementary Table S2. Throughout the administration period, fecal samples were collected from each animal under restraint condition at days 0, 14, and 21, and immediately stored at −80°C for subsequent analysis.

### Fecal DNA extraction and 16s rRNA gene sequencing

Fecal DNAs were extracted using the QIAamp PowerFecal Pro DNA kit (Qiagen, 51804) following the manufacturer’s protocol. Approximately up to 0.25 g of fecal sample was added to a PowerBead Pro tube, along with the solution CD1, and vortexed vigorously for cell lysis. Solution CD2 was added for inhibitor removal, and to bind the DNA, solution CD3 was added and loaded into the MB Spin Column. Then, the column was washed with the solution EA, C5. Lastly, DNA was eluted using solution C6. The V4 sections of the 16s rRNA gene were amplified with the following primers: 515F-TCGTCGGCAGCGTCAGATGTGTATAAGAGACAGGTGCCAGCMGCCGCGGTAA and 806R-GTCTCGTGGGCTCGGAGATGTGTATAAGAGACAGGGACTACHVGGGTWTCTAAT. The amplicons were sequenced utilizing the MiSeq platform (Illumina).

### Gut microbiome analysis

Sequence data was then processed using Mothur v.1.47.0.^1^ and the SILVA reference database following the Mothur Miseq SOP (14). Primers were removed, and sequences were aligned into contigs using the default Mothur settings. Alpha diversity was measured using indices of community richness (Chao and Sobs) and community diversity (Shannon and Invsimpson) (15). All statistical analyses were performed using GraphPad Prism version 7.0 (GraphPad Software, SanDiego, CA, United States), and the significance level used for all tests was *p* < 0.05. The distance between the gut microbiome in each sample was calculated using Jaccard coefficients and visualized using PCoA (16). Statistical significance was also evaluated using analysis of molecular variance (AMOVA) on Mothur v.1.47.0. Linear discriminant analysis (LDA) effect size (LEfSe) (13) was used to identify differentially abundant operational taxonomic unit (OTU). The LDA scores indicate the effect size of each abundant OTU. The cut-off value was an absolute LDA score (log 10) >2.0. The functional capacity of the gut microbiome was predicted using the PICRUSt2 in the MetaCyc pathway (17, 18). Statistical analysis for significant pathways was conducted using STAMP version 2.1.3 (19) and Welch’s *t*-test (two-sided) was used for testing of significance.

## Results

### Changes in the intestinal microbiome of mice subjected to green banana flour

**Figure 1:**
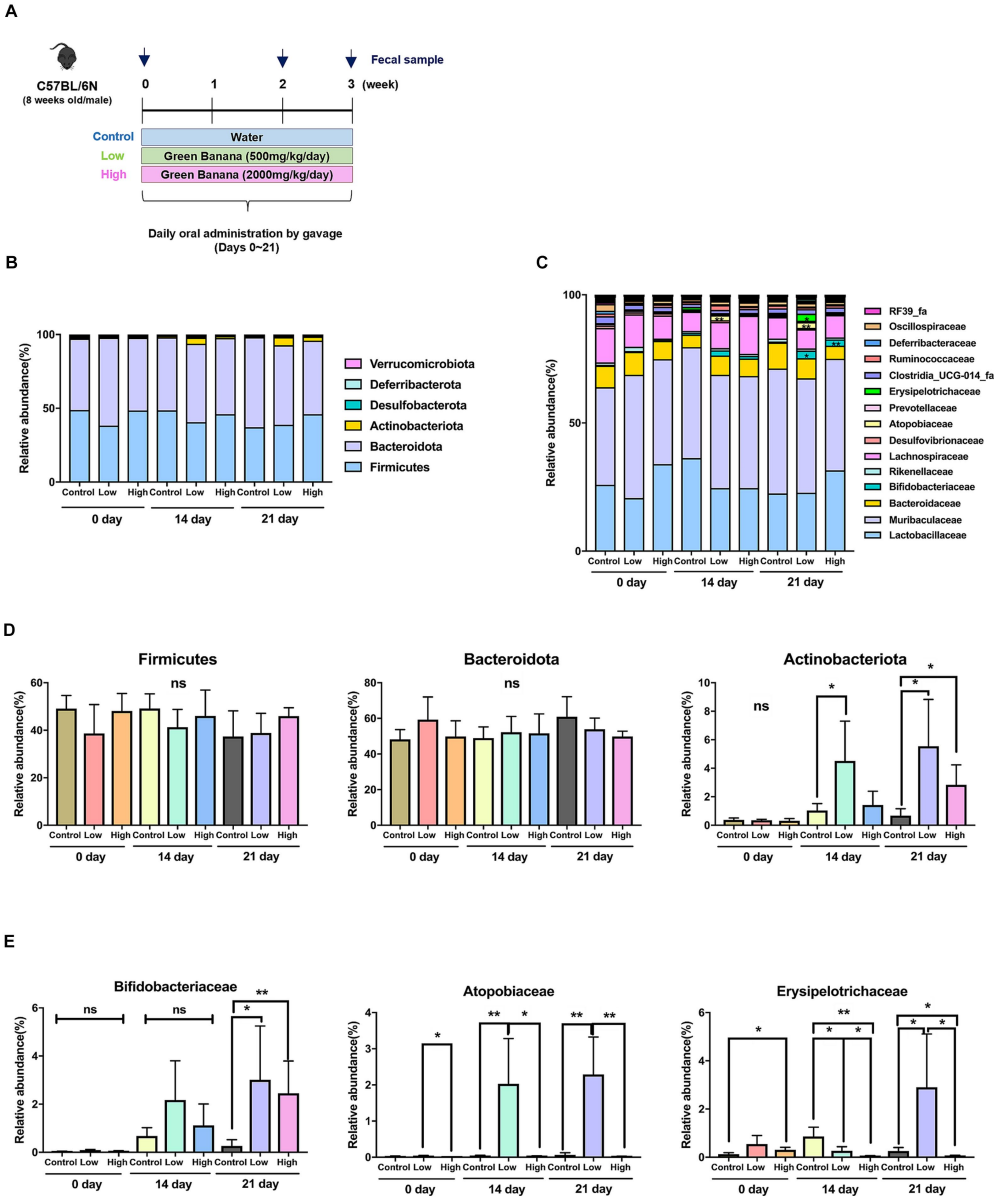
Comparison of intestinal microflora according to green banana flour dosage. **(A)** Schematics of animal study (C57BL/6N) design. Green banana flour was administered to mice daily by oral gavage during weeks 0–3. On days 0, 14, and 21, the mice fecal sample from all the groups was sampled. **(B)** Relative abundance of gut microbiota at the phylum level in green banana-gavaged mice. The six most abundant bacterial phyla were obtained from green banana-gavaged mice on days 0, 14, and 21. The phyla with an abundance of 1% or less are not shown. **(C)** Relative abundance of gut microbiota at the family level in green banana-gavaged mice. The 15 most abundant bacteria families were obtained from green banana-gavaged mice on days 0, 14, and 21. **(D)** Bar chart showing a relative abundance in a main phylum level. **(E)** Bar chart showing relative abundance at a specific family level. Unpaired *t*-tests (two-tailed) were used to analyze variations between the two groups (^*^p < 0.05 and ^**^p < 0.01, ns means no significant).

The quantity of green banana flour to be administered to the mice was established by inversely calculating the human equivalent dose recommended by the Korea Food and Drug Administration (KFDA), adjusted for the weight of the mice. Consequently, the control group received sterilized distilled water, the low-dose group received 500 mg/kg/day, and the high-dose group received 2000 mg/kg/day. These were given to 8 weeks-old C57BL/6N male mice daily for 3 weeks. Fecal samples were collected on days 0, 14, and 21 to analyze the intestinal microbiome (Figure 1A).

An analysis of the intestinal microbiome composition at the phylum level through 16s rRNA sequencing revealed that *Firmicutes* and *Bacteroidota* were the predominant phyla across all groups on day 0. However, by day 14, *Actinobacteriota* significantly increased in the low-dose group (1.1% to 4.2%). By day 21, both the low-dose (0.6% to 5.5%) and high-dose groups (0.6% to 2.8%) demonstrated a notable increase in *Actinobacteriota* relative to the control group (Figures 1B,D).

At the family level, the microbiome comprised microorganisms from families such as *Lactobacillaceae, Muribaculaceae, Bacteroidaceae, Bifidobacteriaceae, Rikenellaceae,* and *Lachnospiraceae,* among others (Figure 1C). There were no significant increases in the low-dose and high-dose groups compared to the control group on day 21 at the five major families (Supplementary Figure S1). Comparisons of relative abundance were made at the level of three specific families, which were almost absent in the control group but rapidly increased in the low-dose or high-dose groups. Microorganisms from the *Bifidobacteriaceae* family were almost nonexistent on day 0, but their presence significantly increased in both the low-dose and high-dose groups compared to the control group by day 21. On days 14 and 21, microorganisms from the *Atopobiaceae* family increased in the low-dose group relative to the control group, but their presence decreased in the high-dose group. Microorganisms from the *Erysipelotrichaceae* family were significantly low in the low-dose group on days 0 and 14, but their numbers increased about threefold on day 21 (Figure 1E; Supplementary Table S3).

### Relation between green banana flour and the alpha and beta diversity of intestinal microbiome

**Figure 2:**
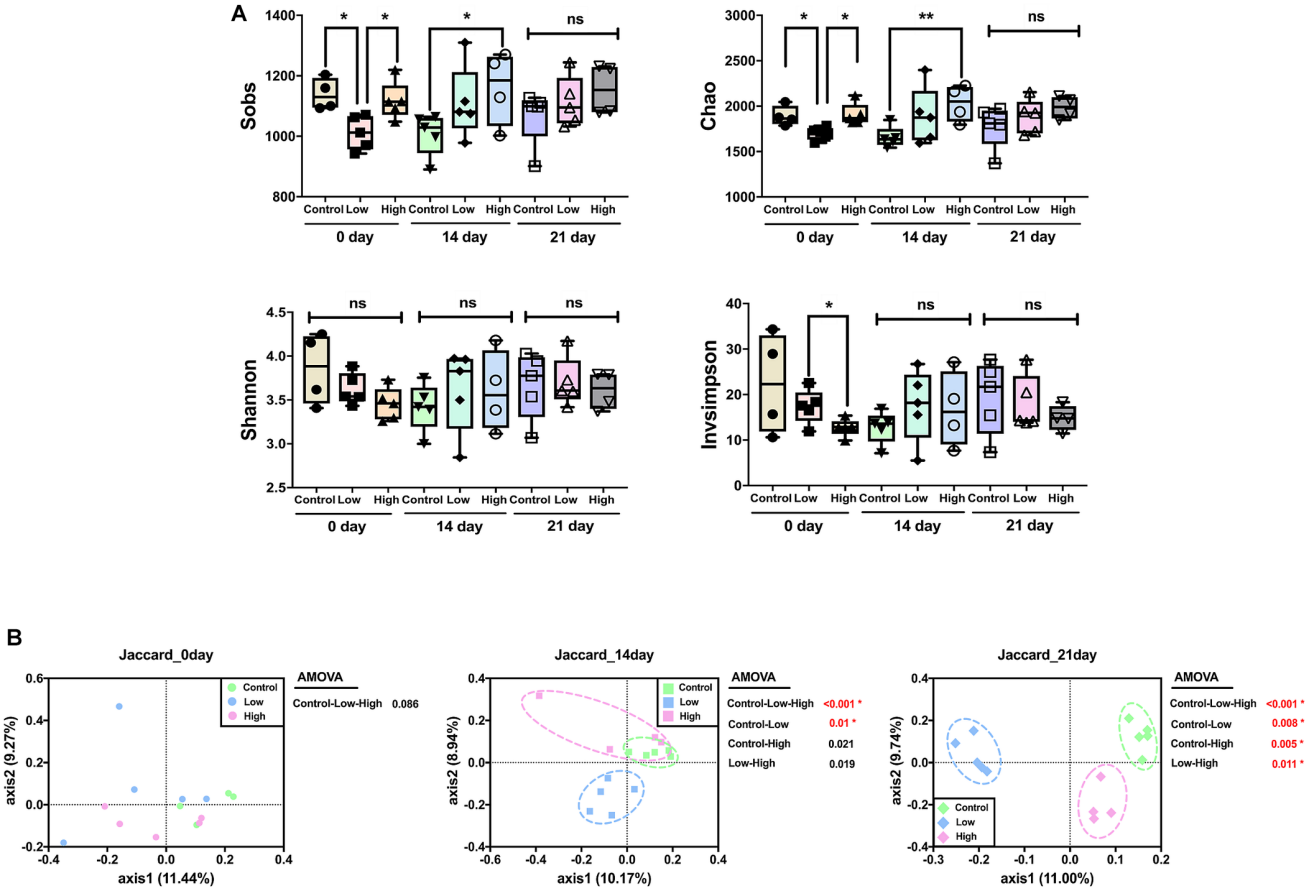
Effects of green banana flour on the diversity of the gut microbiota. **(A)** Alpha diversity. Sobs, Chao, Shannon, and Invsimpson indices reflect community richness and diversity. Unpaired *t*-tests (two-tailed) were used to analyze variations between the two groups (^*^p < 0.05 and ^**^p < 0.01, ns means no significant). **(B)** Beta diversity. Principal-coordinate analysis plot based on Jaccard distances. The principal coordinate with the largest contribution rate was selected for graphical display. Statistics analysis uses AMOVA in Mothur.

Alpha diversity was analyzed using Sobs and Chao, which are measures of community richness, and Shannon and Invsimpson, which are measures of community diversity. On day 14, richness of the high-dose group increased significantly compared to the control group, and there was no significant difference among all groups on the 21st day. There was no significant difference in Shannon and Invsimpson, which are measures of community diversity (Figure 2A). Principal coordinate analysis (PCoA) was performed using the Jaccard distance, identifying differences in microbial community composition. The analysis showed no significant difference on day 0 but a significant difference between the control and low-dose groups on day 14. On day 21, significant differences were observed between control and each of the low-dose, high-dose, and low-dose versus high-dose groups. Significant differences between the groups were analyzed using analysis of molecular variance (AMOVA) (Figure 2B).

### Differential taxonomic composition among groups

**Figure 3:**
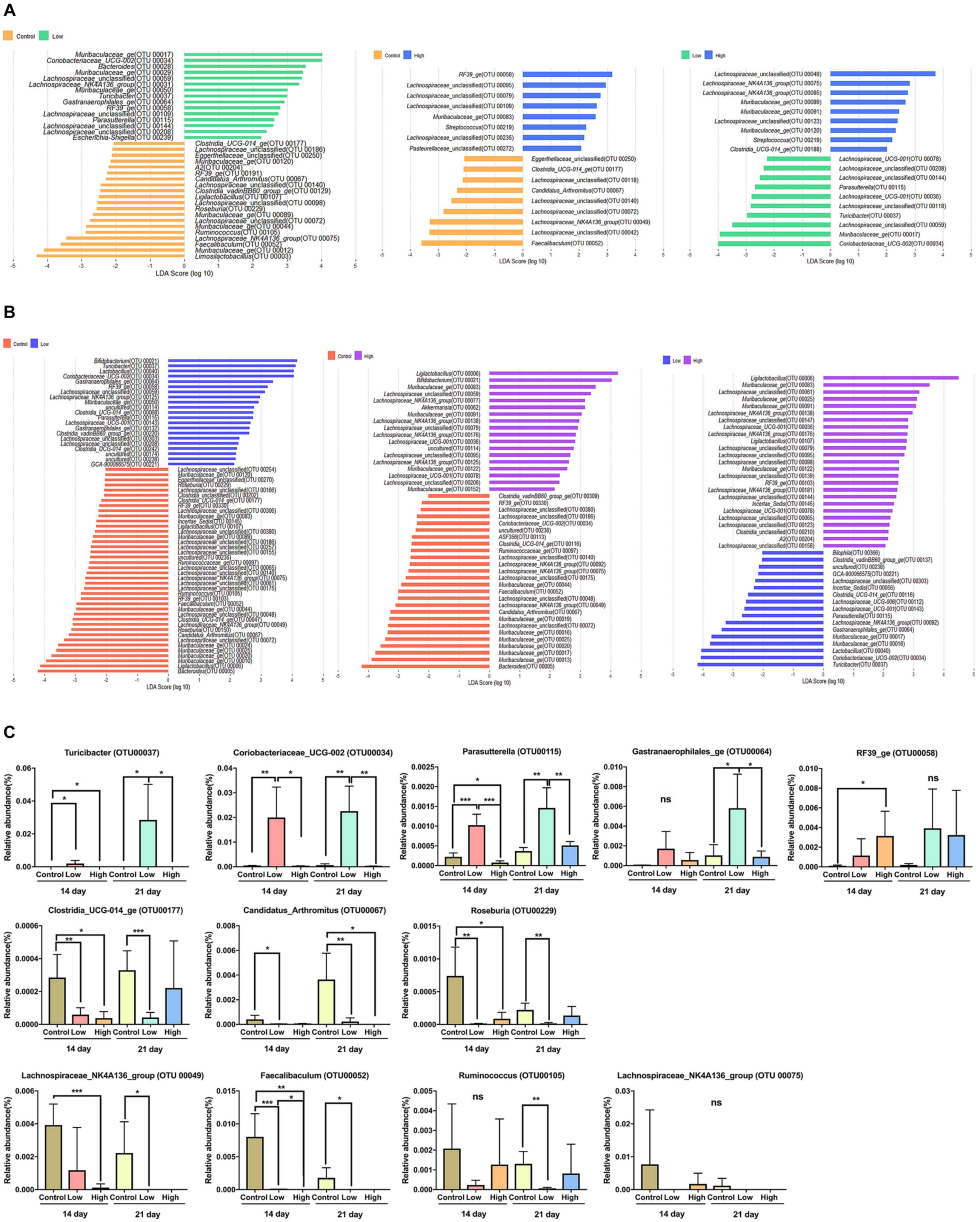
Differential bacterial taxa between two groups. **(A)** A linear discriminant analysis effect size (LEfSe) bar plot showing enriched taxa in different groups on day 14. **(B)** A linear discriminant analysis effect size (LEfSe) bar plot showing enriched taxa in different groups on day 21. The LDA threshold score was 2 or more, and the *p*-value was 0.05 or less, indicating a difference in bacteria at the genus level. **(C)** Box plot showing relative abundance for OTUs that were significantly different in LEfSe on days 14 and 21. Unpaired *t*-tests (two-tailed) were used to analyze variation between the two groups (^*^p < 0.05, ^**^p < 0.01, and ^***^p < 0.001, ns means no significant).

To distinguish the presence and effect size of operational taxonomic units (OTUs) among different groups, a linear discriminant analysis effect size (LEfSe) analysis was performed. A logarithmic LDA score cut-off of 2.0 was utilized to identify taxonomic differences among the control, low-dose, and high-dose groups. The OTUs that displayed differences between groups on days 14 and 21 are presented. On both days 14 and 21, when compared to the control and low-dose groups, the bacteria that were differential ranking them according to the effect size were *Coriobacteriaceae_UCG-002* (OTU00034), *Turicibacter* (OTU00037), *Gastranaerophilales_ge* (OTU00064), *RF39_ge* (OTU00058), *Parasutterella* (OTU00115), *Clostridia_UCG-014_ge* (OTU00177), *Muribaculaceae_ge* (OTU00120), *Candidatus_Arthromitus* (OTU00067), *Ligilactobacillus* (OTU00107), *Roseburia* (OTU00229), *Muribaculaceae_ge* (OTU00044), *Ruminococcus* (OTU00105), *Lachnospiraceae_NK4A136_group* (OTU00075), and *Faecalibaculum* (OTU00052). When comparing the control and high-dose groups, *Muribaculaceae_ge* (OTU00083), *Candidatus_Arthromitus* (OTU00067), *Lachnospiraceae_NK4A136_group* (OTU00049), and *Faecalibaculum* (OTU00052) were differential ranked according to the effect size. In comparing the low-dose and high-dose groups, *Muribaculaceae_ge* (OTU00091), *Parasutterella* (OTU00115), *Turicibacter* (OTU00037), *Muribaculaceae_ge* (OTU00017), and *Coriobacteriaceae_UCG-002* (OTU00034) were showed significant differences of effect size (Figures 3A,B). Based on the rankings by LEfSe, the relative abundance of each significant OTUs was analyzed, and it concluded that consumption of green bananas led to an increase in *Coriobacteriaceae_UCG-002, Turicibacter, Gastranaerophilales_ge, RF39_ge*, and *Parasutterella*, while there was a decrease in *Clostridia_UCG-014_ge, Candidatus_Arthromitus, Roseburia, Ruminococcus, Lachnospiraceae_NK4A136_group,* and *Faecalibaculum* (Figure 3C).

### Analysis of metabolic pathway change due to consumption of green banana flour

**Figure 4:**
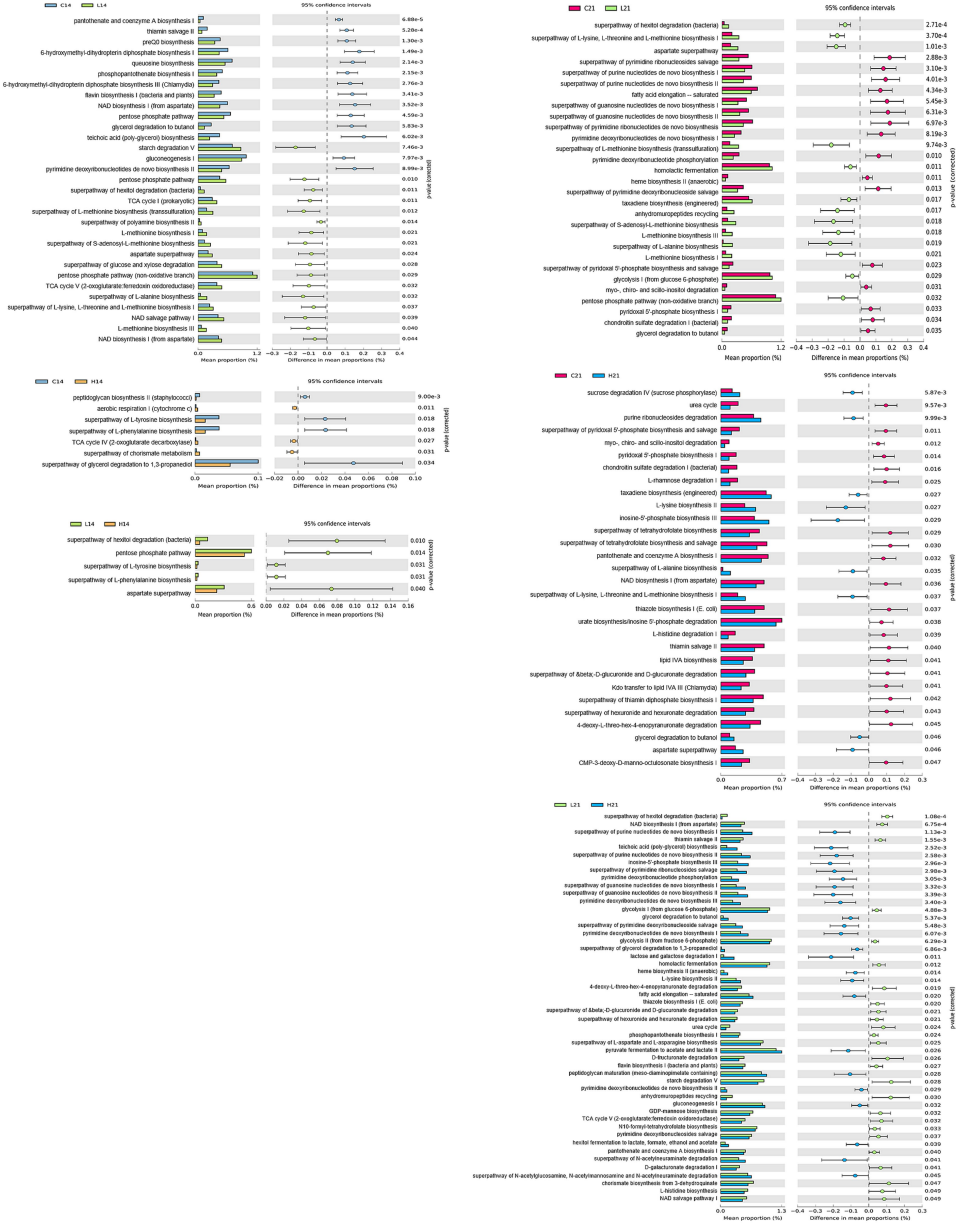
Comparison of PICRUSt-predicted functional pathways between two groups (control vs. low-dose, control vs. high-dose, low-dose vs. high-dose) on days 14 and 21. The extended error bar plots show the significantly different MetaCyc pathways between the two groups. Bar plots on the left display each group’s mean proportion of pathway. Dot plots on the right show the difference in the mean proportions between the two groups using Welch’s *t*-test (two-sided).

To gain deeper insight into the metabolic signatures associated with gut microbiota alterations induced by green banana flour, a phylogenetic investigation of communities by reconstruction of unobserved states (PICRUSt2) was employed with the MetaCyc database. Fourteen days post green banana flour administration, the superpathway of glycerol degradation to 1,3-propanediol showed a decrease in both the low-dose and high-dose groups relative to the control group, with no pathways exhibiting an increase. On day 21, several pathways—namely, the aspartate superpathway, superpathway of L-alanine biosynthesis, taxadiene biosynthesis (engineered), and the superpathway of L-lysine, L-threonine, and L-methionine biosynthesis I—all demonstrated an increase in both the low-dose and high-dose groups when compared to the control group. In contrast, pathways like the chondroitin sulfate degradation I (bacterial), myo-, chiro-and scillo-inositol degradation, pyridoxal 5′-phosphate biosynthesis I, and the superpathway of pyridoxal 5′-phosphate biosynthesis and salvage showed a decrease in both the low-dose and high-dose groups relative to the control group (Figure 4).

## Discussion

The prevalence of individuals routinely consuming biotic products like probiotics and prebiotics has escalated recently, kindling an upsurge in biotics-related research. Many past studies have demonstrated improvements in index markers and symptom alleviation through prebiotics administration, as evidenced by animal trials, cell experiments, and human application tests. Next-generation sequencing (NGS) has allowed us to analyze microbial communities, thereby enabling us to confirm changes in the intestinal flora, identify valuable microbial target strains for specific diseases, and validate their potential as prebiotics.

In our study, we administered varying doses of green banana flour to normal mice to ascertain the resultant changes in gut microbiota and infer the functional profile of the microbiome. In all groups, the body weight of mice tended to increase over time and there was no significant difference between groups (Supplementary Figure S2). Although control animals were given distilled water instead of standard diet used for feeding via oral gavage, considering that the absolute intake quantity of green banana flour used in this study ranged from only 10 to 40 mg (approximately 0.01% of daily food intake) per animal, the stress associated with force-feeding appeared to be minimal. Following the ingestion of green banana flour, a significant increase in *Actinobacteriota* was observed (Figures 1B,D). *Actinobacteriota,* one of the largest bacterial phyla, is a gram-positive bacterium with high G+C DNA, ubiquitously found in aquatic and terrestrial ecosystems. The phylum is known for its ability to produce short chain fatty acids (SCFAs) from resistant starch, which stimulate salt and water absorption, provide energy, and induce trophic effects on the mucous membranes of the colon and small intestine (20). Especially, *Bifidobacteriaceae* and *Atopobiaceae*, members of the *Actinobacteriota* phylum increased by green banana flour consumption (Figures 1C,E). They are known to promote health through dietary fiber digestion, infection prevention, and the production of critical chemicals. They also produce beneficial lactates and SCFAs, including acetate and butyrate (21, 22). Although there was an increase in proportions of SCFAs producing bacteria, as SCFAs were not determined, it remains unclear whether this bacterial changes directly influenced SCFA production. Therefore, additional experiments, such as measuring SCFA production levels, are required to substantiate this.

The consumption of green banana flour had no effect on the diversity or richness of the gut microbiome. Although, changes in the composition of the microbial community were significant in all groups on day 21 after ingestion (Figure 2). Based on LEfSe analysis, specific bacteria that showed consistent increase in the low and high-dose groups compared to the control group were *Coriobacteriaceae_UCG-002, Turicibacter, Gastranaerophilales_ge, RF39_ge,* and *Parasutterella*. Conversely, bacteria that consistently decreased were *Clostridia_UCG-014_ge, Candidatus_Arthromitus, Roseburia, Ruminococcus, Lachnospiraceae_NK4A136_group,* and *Faecalibaculum*. Altered microbes in our study show associations with various disease models. *Turicibacter*, typically reduced in obese mice due to a high-fat diet, increased in our study; *Candidatus_Arthromitus*, usually elevated, decreased (23, 24). *Coriobacteriaceae_UCG-002,* elevated in our study, correlates positively with synthesizing polyunsaturated fatty acids (PUFAs), lowering low-density lipoprotein (LDL) cholesterol. *Parasutterella* improves LDL levels; decreased *Roseburia* is prominent in mild strok group (25–27). Diminished *Lachnospiraceae_NK4A136_group* is linked to intestinal diseases. *Clostridia_UCG-014_ge, Ruminococcus*, and *Faecalibaculum* relate to impaired intestinal barrier in Crohn’s patients (28–31). Increased *RF39_ge* in our study correlates negatively with diabetes and cognitive impairment (32) (Figure 3).

Differences in metabolic pathways related to altered microbiomes were observed between groups. Green banana flour supplements increased pathways commonly increased pathways like aspartate superpathway, superpathway of L-alanine biosynthesis, taxadiene biosynthesis (engineered) and the superpathway of L-lysine, L-threonine, and L-methionine biosynthesis I. In contrast, chondroitin sulfate degradation I (bacterial), myo-, chiro-and scillo-inositol degradation, pyridoxal 5′-phosphate biosynthesis I, and the superpathway of pyridoxal 5′-phosphate biosynthesis and salvage were reduced. It was predicted that consuming green banana flour may have various beneficial effects on metabolic syndrome, diabetes, and cardiovascular health, as indicated by a taxonomic-based analysis of metabolic pathways. After consuming green banana flour, myo-, chiro-and scillo-inositol degradation pathways decreased. These pathways break down inositols, affecting insulin action and playing roles in signal transduction. Inositol deficiency relates to conditions like metabolic syndrome, neural tube defects, and diabetes (33–35). Similarly, reduced pyridoxal 5′-phosphate (PLP) biosynthesis I, the superpathway of pyridoxal 5′-phosphate biosynthesis and salvage pathway synthesize metabolically active vitamin B6 coenzyme PLP. PLP sources include meat, dairy, beans, nuts, and produce; it associates with cardiovascular health (36, 37). Increased pathways post-green banana flour ingestion were aspartate superpathway, superpathway of L-alanine biosynthesis, taxadiene biosynthesis (engineered), superpathway of L-lysine, L-threonine, and L-methionine biosynthesis I. Aspartate superpathway increased in weight-loss group due to lifestyle changes (38). Synthesis of essential amino acids like L-alanine, L-lysine, L-threonine, and L-methionine also rose. L-alanine is an essential component of cellular proteins and peptidoglycan, synthesized for cell biosynthesis and involved in transamination (39). The biosynthesis pathways of L-lysine, L-threonine, and L-methionine stem from TCA cycle’s oxaloacetate. The taxadiene biosynthesis (engineered) is essential for membrane synthesis protein assembly (40) (Figure 4).

In summation, our investigation did not ascertain definitive therapeutic or prophylactic effects of green banana flour consumption within a specific disease model. Nevertheless, it did substantiate conspicuous alterations in the intestinal microbiota and associated biochemical pathways resulting from the administration of varied quantities of green bananas to murine subjects. Our findings revealed an augmented prevalence of beneficial bacterial taxa such as *Coriobacteriaceae_UCG-002* and *Parasutterella*, coupled with heightened engagement of pathways implicated in amino acid biosynthesis and secondary metabolite production, subsequent to green banana flour ingestion. Beyond its conventional merits encompassing gastrointestinal, renal, hepatic, and metabolic dimensions, our data underscores the latent prebiotic attributes of green banana flour. While acknowledging the intrinsic limitations of our study, the results here reported suggest a causal nexus between changes in gut microbial ecology and concomitant metabolic reprogramming, thereby shedding light on the potential salutary effects of green banana flour.

## Data availability statement

The original contributions presented in the study are publicly available. This data can be found at: https://www.ncbi.nlm.nih.gov/bioproject/991718.

## Ethics statement

The animal study was approved by Institute Animal Care and Use Committee of Korea Research Institute of Bioscience and Biotechnology. The study was conducted in accordance with the local legislation and institutional requirements.

## Author contributions

J-SK: designed the study. GB, Y-JK, S-CJ, and HS: performed the experiments. YL: provided critical materials and scientific insight. GB and J-SK: analyzed and visualized the data. GB, HS, and J-SK: wrote the paper. All authors contributed to the article and approved the submitted version.

## Footnotes

^1^http://www.mothur.org/
