# Supplementary material for: Prebiotic potential of green banana flour: impact on gut microbiota modulation and microbial metabolic activity in a murine model
Source: Front Nutr. 2023 Oct 31;10:1249358. doi: 10.3389/fnut.2023.1249358 (PMC10644147; doi:10.3389/fnut.2023.1249358)
Supplement: Supplementary file 1 [file Data_Sheet_1.docx]

**Table S1.** The nutritional information of the standard diet used for feeding.

| Nutritional composition: | Per 100 g |
| --- | --- |
| Energy Density (cal) | 310 |
| Total Fat (g) | 6.0 |
| - of which saturated fat (g) | 0.9 |
| - of which monounsaturated fat (g) | 1.3 |
| - of which polyunsaturated fat (g) | 3.4 |
| Cholesterol (mg) | 0 |
| Total Carbohydrates (g) | 62.7 |
| - Carbohydrate (available) | 44.2 |
| - Crude fiber | 3.8 |
| - Neutral Detergent fiber (insoluble) | 14.7 |
| Protein (g) | 18.4 |
| Ash (g) | 5.5 |
| Sodium (g) | 0.2 |
| Vitamin D_3_ (IU) | 200 |
| Calcium (g) | 1.0 |
| Iron (mg) | 20.0 |
| Potassium (mg) | 600 |
| Magnesium (mg) | 200 |
| Manganese (mg) | 10.0 |

**Table S2.** The nutritional information of the green banana flour

| Nutritional composition: | Per 100 g |
| --- | --- |
| Energy (cal) | 209 |
| Total Fat (g) | 0.5 |
| - of which saturated fat (g) | 0 |
| - of which monounsaturated fat (g) | 0.067 |
| - of which polyunsaturated fat (g) | 0.139 |
| - of which trans fatty acids (g) | < 0.007 |
| Cholesterol (mg) | 0 |
| Total Carbohydrates (g) | 89 |
| - Total sugars (g) | 2 |
| - Added Sugars (g) | 0 |
| - Sugar Alcohol (g) | 0 |
| - Dietary fiber (g) | 41 |
| Protein (g) | 4 |
| Ash (g) | 2.75 |
| Sodium (g) | 0 |
| Moisture (g) | 3.75 |
| Vitamin D (mcg) | 0.1 |
| Calcium (mg) | 18.4 |
| Iron (mg) | 0.9 |
| Potassium (mg) | 1200 |
| Magnesium (mg) | 98.5 |
| Manganese (mg) | 0.75 |

**Table S3.** Comparison of relative abundance by group and age at main family levels

Unpaired t-tests (two-tailed) were used to analyze variations between the two groups. (*p< 0.05, **p< 0.01, ns means no significant)

| Family | Group | | | Age | Statistics |
| --- | --- | --- | --- | --- | --- |
| *Lactobacillaceae* | Control (C) | Low (L) | High (H) | Day | Unpaired t-tests (two tailed) |
|  | 17.04804 | 17.67295547 | 22.90460203 | 0 | C vs L ns  C vs H ns  L vs H * |
|  | 31.03059 | 15.51122315 | 34.01205371 |  |  |
|  | 15.13364 | 35.29056574 | 40.73492386 |  |  |
|  | 42.73349863 | 15.33433578 | 36.89389375 |  |  |
|  | X | 18.34522604 | 33.23700715 |  |  |
|  | 31.25718956 | 23.46583396 | 42.18205814 | 14 | C vs L ns  C vs H ns  L vs H ns |
|  | 36.58109531 | 21.90232235 | 7.359806831 |  |  |
|  | 33.67849 | 16.35351234 | 22.56035695 |  |  |
|  | 50.54959 | 18.70651194 | X |  |  |
|  | 31.84384 | 48.57601072 | 30.76944633 |  |  |
|  | 12.01093342 | 19.12340542 | 25.61016456 | 21 | C vs L ns  C vs H ns  L vs H * |
|  | 24.15088608 | 17.41434871 | X |  |  |
|  | 46.51831097 | 18.11178891 | 36.98552108 |  |  |
|  | 17.05793971 | 29.43015166 | 32.85474386 |  |  |
|  | 15.84016918 | 28.62120943 | 30.86794049 |  |  |
| *Muribaculaceae* | 31.87986224 | 44.09393985 | 51.59583046 | 0 | C vs L ns  C vs H ns  L vs H ns |
|  | 46.13520907 | 37.26184128 | 36.40677449 |  |  |
|  | 39.00368913 | 41.6745186 | 35.76902393 |  |  |
|  | 34.57337475 | 57.87522632 | 29.12663855 |  |  |
|  | X | 57.86276882 | 54.15982048 |  |  |
|  | 41.16062239 | 51.80617202 | 40.70169007 | 14 | C vs L ns  C vs H ns  L vs H ns |
|  | 44.84597156 | 45.14763159 | 28.56913461 |  |  |
|  | 40.66178866 | 38.31748516 | 51.81474393 |  |  |
|  | 37.02098099 | 49.09556393 | X |  |  |
|  | 50.14881832 | 32.8636636 | 54.40559343 |  |  |
|  | 59.00715839 | 51.42313212 | 45.05606524 | 21 | C vs L ns  C vs H ns  L vs H ns |
|  | 53.91963294 | 38.51693238 | X |  |  |
|  | 39.55583095 | 50.56036317 | 44.5698275 |  |  |
|  | 43.77003877 | 40.89698895 | 46.88794182 |  |  |
|  | 47.84433265 | 42.36727629 | 38.11990026 |  |  |
| *Bacteroidaceae* | 9.838917847 | 14.05496 | 3.761966896 | 0 | C vs L ns  C vs H ns  L vs H ns |
|  | 6.629009912 | 4.410077 | 11.45254806 |  |  |
|  | 9.735516683 | 6.047536 | 11.55430345 |  |  |
|  | 7.419048585 | 12.53874 | 6.492797781 |  |  |
|  | X | 7.24015 | 2.580987514 |  |  |
|  | 4.203245 | 9.313013 | 4.528357 | 14 | C vs L ns  C vs H ns  L vs H ns |
|  | 5.052001 | 2.843544 | 9.251054 |  |  |
|  | 7.516003 | 14.08467 | 8.531833 |  |  |
|  | 2.458627 | 5.391942 | X |  |  |
|  | 5.118299 | 5.035952 | 4.139023 |  |  |
|  | 12.87344 | 4.533687 | 3.908548 | 21 | C vs L ns  C vs H *  L vs H ns |
|  | 8.876828 | 4.004127 | X |  |  |
|  | 3.80139 | 7.07689 | 3.627812 |  |  |
|  | 12.17016 | 10.60706 | 5.310202 |  |  |
|  | 11.98153 | 12.15333 | 7.299392 |  |  |
| *Bifidobacteriaceae* | 0.004991479 | 0.071239324 | 0.076969315 | 0 | C vs L ns  C vs H ns  L vs H ns |
|  | 0.061813919 | 0.034744106 | 0.016020751 |  |  |
|  | 0.005751423 | 0.046448051 | 0.056222347 |  |  |
|  | 0.008295619 | 0.068739068 | 0.02387476 |  |  |
|  | X | 0.138268191 | 0.014065327 |  |  |
|  | 0.426076164 | 1.563952499 | 1.82644719 | 14 | C vs L ns  C vs H ns  L vs H ns |
|  | 0.552922591 | 2.514666193 | 0.264060948 |  |  |
|  | 0.382139807 | 0.349052226 | 0.444462379 |  |  |
|  | 0.830094441 | 1.707737118 | X |  |  |
|  | 1.205001347 | 4.736350499 | 1.94130546 |  |  |
|  | 0.225001875 | 6.895358145 | 4.003203728 | 21 | C vs L *  C vs H **  L vs H ns |
|  | 0.287767484 | 1.219046811 | X |  |  |
|  | 0.691488406 | 2.189672294 | 2.502443943 |  |  |
|  | 0.080490455 | 2.734507156 | 2.579164632 |  |  |
|  | 0.025608618 | 2.013422819 | 0.733272227 |  |  |
| *Rikenellaceae* | 1.608682 | 2.547006 | 1.21978 | 0 | C vs L ns  C vs H ns  L vs H * |
|  | 1.052258 | 0.54621 | 0.720171 |  |  |
|  | 1.170004 | 2.010537 | 0.761446 |  |  |
|  | 1.126361 | 1.847976 | 0.374038 |  |  |
|  | X | 2.757134 | 1.175094 |  |  |
|  | 0.867288 | 0.985244 | 0.645232 | 14 | C vs L ns  C vs H ns  L vs H ns |
|  | 0.758294 | 0.1705 | 0.906966 |  |  |
|  | 0.819752 | 1.885852 | 1.080891 |  |  |
|  | 0.184661 | 0.826996 | X |  |  |
|  | 0.802896 | 0.540164 | 0.458879 |  |  |
|  | 1.530846 | 0.855249 | 0.625455 | 21 | C vs L ns  C vs H ns  L vs H ns |
|  | 1.227009 | 0.476312 | X |  |  |
|  | 0.30174 | 0.870336 | 0.72587 |  |  |
|  | 1.608468 | 1.085566 | 1.018285 |  |  |
|  | 1.652995 | 0.860275 | 0.687763 |  |  |
| *Lachnospiraceae* | 21.56533 | 8.896111 | 15.48843 | 0 | C vs L ns  C vs H ns  L vs H ns |
|  | 7.540588 | 36.21871 | 5.690418 |  |  |
|  | 19.13499 | 9.388478 | 5.0154 |  |  |
|  | 4.123844 | 3.399515 | 13.68365 |  |  |
|  | X | 8.836819 | 5.114025 |  |  |
|  | 14.13695 | 2.695183 | 7.137882 | 14 | C vs L ns  C vs H ns  L vs H ns |
|  | 5.546998 | 15.29163 | 38.63021 |  |  |
|  | 11.43073 | 16.31611 | 7.413471 |  |  |
|  | 3.258824 | 13.41802 | X |  |  |
|  | 4.007254 | 1.794967 | 3.352272 |  |  |
|  | 5.047542 | 4.65772 | 12.91685 | 21 | C vs L ns  C vs H ns  L vs H ns |
|  | 3.773751 | 19.58107 | X |  |  |
|  | 3.179051 | 4.907079 | 5.118041 |  |  |
|  | 15.11275 | 5.05494 | 6.224116 |  |  |
|  | 12.85883 | 3.806189 | 10.63758 |  |  |
| *Desulfovibrionaceae* | 0.946954841 | 0.301766575 | 0.425163835 | 0 | C vs L ns  C vs H ns  L vs H ns |
|  | 0.561298803 | 2.08868635 | 0.273878547 |  |  |
|  | 1.832239193 | 0.805541916 | 0.201667115 |  |  |
|  | 0.323529141 | 0.470126124 | 2.327220637 |  |  |
|  | X | 0.257606808 | 0.861820948 |  |  |
|  | 0.287582491 | 0.261858937 | 0.149576566 | 14 | C vs L ns  C vs H ns  L vs H ns |
|  | 0.448262243 | 2.596279716 | 0.719030825 |  |  |
|  | 0.239497759 | 0.344204279 | 0.651416976 |  |  |
|  | 0.536395772 | 0.45481865 | X |  |  |
|  | 0.332459477 | 0.283740439 | 0.244502203 |  |  |
|  | 0.261668847 | 0.19830825 | 0.444881316 | 21 | C vs L ns  C vs H ns  L vs H ns |
|  | 0.40527254 | 1.335651289 | X |  |  |
|  | 0.760637247 | 0.363172081 | 0.261713608 |  |  |
|  | 0.311900514 | 0.337584396 | 0.320472077 |  |  |
|  | 0.646824118 | 0.45965417 | 0.884541673 |  |  |
| *Atopobiaceae* | 0.001426137 | 0.037620767 | 0.004398247 | 0 | C vs L ns  C vs H ns  L vs H * |
|  | 0.044051298 | 0.048480147 | 0.003051572 |  |  |
|  | 0.003286528 | 0.003981262 | 0.00244445 |  |  |
|  | 0.001843471 | 0.044803142 | 0.00170534 |  |  |
|  | X | 0.008230249 | 0.005753997 |  |  |
|  | 0.018163105 | 1.537766605 | 0.015397588 | 14 | C vs L **  C vs H ns  L vs H * |
|  | 0.053317536 | 3.644326648 | 0.014868297 |  |  |
|  | 0.020251649 | 0.530503979 | 0.020753107 |  |  |
|  | 0.051001565 | 1.466746804 | X |  |  |
|  | 0.040736141 | 2.964294526 | 0.04553766 |  |  |
|  | 0.040000333 | 3.874582651 | 0.02621232 | 21 | C vs L **  C vs H ns  L vs H ** |
|  | 0.059951559 | 1.123643148 | X |  |  |
|  | 0.167932899 | 1.798836714 | 0.01308568 |  |  |
|  | 0.027500906 | 2.60506303 | 0.022747496 |  |  |
|  | 0.032217293 | 2.052969125 | 0.030766667 |  |  |
| *Prevotellaceae* | 0.325159193 | 0.727601636 | 0.211115835 | 0 | C vs L ns  C vs H ns  L vs H ns |
|  | 0.884578493 | 0.380569157 | 0.572169667 |  |  |
|  | 0.876681264 | 0.422677266 | 0.292722873 |  |  |
|  | 0.428606981 | 0.498971983 | 0.312077218 |  |  |
|  | X | 1.278980766 | 0.368895169 |  |  |
|  | 0.385209178 | 0.303101719 | 0.087986215 | 14 | C vs L ns  C vs H ns  L vs H ns |
|  | 0.717482886 | 0.34988768 | 0.337807699 |  |  |
|  | 0.686795045 | 0.714725987 | 0.537274886 |  |  |
|  | 0.277870597 | 0.497006403 | X |  |  |
|  | 0.814065795 | 0.413274118 | 0.229790036 |  |  |
|  | 1.128342736 | 1.119900774 | 0.100480559 | 21 | C vs L ns  C vs H ns  L vs H ns |
|  | 0.816939913 | 0.224021936 | X |  |  |
|  | 0.310720764 | 0.288693432 | 0.29712192 |  |  |
|  | 0.432636197 | 0.367003516 | 0.472345066 |  |  |
|  | 1.189561597 | 0.334137633 | 0.364713197 |  |  |
| *Erysipelotrichaceae* | 0.087707414 | 0.814049355 | 0.423697752 | 0 | C vs L ns  C vs H *  L vs H ns |
|  | 0.215993463 | 0.452481376 | 0.265486726 |  |  |
|  | 0.115850101 | 0.197735989 | 0.411889804 |  |  |
|  | 0.082034454 | 1.014514991 | 0.243295172 |  |  |
|  | X | 0.264191008 | 0.188603249 |  |  |
|  | 0.43440092 | 0.157115362 | 0.024929428 | 14 | C vs L *  C vs H **  L vs H * |
|  | 1.36716693 | 0.547861079 | 0.061852114 |  |  |
|  | 0.515096283 | 0.125354073 | 0.053612194 |  |  |
|  | 0.96551239 | 0.191867588 | X |  |  |
|  | 1.020374641 | 0.314581791 | 0.062351565 |  |  |
|  | 0.168334736 | 2.306865792 | 0.071355759 | 21 | C vs L *  C vs H *  L vs H * |
|  | 0.242204299 | 3.692475124 | X |  |  |
|  | 0.466979184 | 6.349836856 | 0.076204845 |  |  |
|  | 0.320620313 | 1.053939956 | 0.03010698 |  |  |
|  | 0.091695373 | 1.117613008 | 0.039099306 |  |  |
| *Clostridia_UCG-014_fa* | 3.642353411 | 4.356004514 | 0.659003944 | 0 | C vs L ns  C vs H ns  L vs H ns |
|  | 1.654055206 | 0.117160356 | 4.044095209 |  |  |
|  | 2.935690869 | 0.684113439 | 1.736781637 |  |  |
|  | 3.598455171 | 3.233804892 | 2.249911891 |  |  |
|  | X | 0.576117462 | 0.702627019 |  |  |
|  | 1.751982806 | 1.460518219 | 0.406203028 | 14 | C vs L ns  C vs H ns  L vs H ns |
|  | 1.224986835 | 0.850073533 | 2.488358124 |  |  |
|  | 0.695600109 | 1.090788207 | 2.388913229 |  |  |
|  | 0.871423295 | 2.141172935 | X |  |  |
|  | 1.568341448 | 0.879419125 | 1.381542536 |  |  |
|  | 2.575854799 | 1.197781832 | 2.198922382 | 21 | C vs L ns  C vs H ns  L vs H ns |
|  | 1.394073589 | 1.804189281 | X |  |  |
|  | 1.138710778 | 1.80663924 | 1.699598962 |  |  |
|  | 2.35367506 | 1.942397364 | 1.039025337 |  |  |
|  | 1.860342164 | 2.079906464 | 3.128585438 |  |  |
| *Ruminococcaceae* | 1.873230699 | 0.437841689 | 0.707384656 | 0 | C vs L ns  C vs H ns  L vs H ns |
|  | 0.655085438 | 0.335321019 | 2.150595056 |  |  |
|  | 1.299821706 | 0.462489881 | 0.224889389 |  |  |
|  | 0.316155257 | 0.415503115 | 1.46829773 |  |  |
|  | X | 0.512744541 | 0.181570586 |  |  |
|  | 1.200278501 | 2.369823376 | 0.256626462 | 14 | C vs L ns  C vs H ns  L vs H ns |
|  | 0.724065298 | 0.580991321 | 2.053014398 |  |  |
|  | 0.5309454 | 4.936595771 | 0.415062144 |  |  |
|  | 0.32623415 | 0.904436071 | X |  |  |
|  | 0.806838415 | 0.18328575 | 0.495309621 |  |  |
|  | 1.914182618 | 0.436278151 | 0.785641474 | 21 | C vs L ns  C vs H ns  L vs H ns |
|  | 1.622688867 | 1.656490276 | X |  |  |
|  | 0.537924098 | 1.002269826 | 1.55334724 |  |  |
|  | 0.913566666 | 0.315520056 | 0.714538995 |  |  |
|  | 0.692258763 | 0.280262954 | 0.731349311 |  |  |
| *Deferribacteraceae* | 2.43584167 | 0.027215023 | 0.075503233 | 0 | C vs L ns  C vs H ns  L vs H ns |
|  | 0.061813919 | 0.257752784 | 0.019835215 |  |  |
|  | 1.267778061 | 0.220296471 | 0.027500061 |  |  |
|  | 0.049773714 | 0.193328628 | 2.141338578 |  |  |
|  | X | 0.009053274 | 0.095899957 |  |  |
|  | 0.021190289 | 0.011129005 | 0.087252997 | 14 | C vs L ns  C vs H ns  L vs H ns |
|  | 0.013823065 | 0.370089048 | 0.277145049 |  |  |
|  | 0.05723292 | 0.038783581 | 0.222519427 |  |  |
|  | 0.111675841 | 0.422455443 | X |  |  |
|  | 0.013140691 | 0.027316626 | 0.009808111 |  |  |
|  | 0.021666847 | 0.001442242 | 0.287607398 | 21 | C vs L ns  C vs H ns  L vs H ns |
|  | 0.047161893 | 0.1597128 | X |  |  |
|  | 0.01436859 | 0.027663498 | 0.033099074 |  |  |
|  | 0.814295105 | 0.034567465 | 0.07359484 |  |  |
|  | 0.026434702 | 0.034961227 | 0.554440976 |  |  |
| *Oscillospiraceae* | 4.530123575 | 1.243086184 | 0.708850738 | 0 | C vs L ns  C vs H ns  L vs H ns |
|  | 0.969839071 | 0.751442284 | 1.081019225 |  |  |
|  | 4.245372158 | 1.298554802 | 0.714390476 |  |  |
|  | 0.610188864 | 0.775155737 | 2.274923544 |  |  |
|  | X | 0.412335498 | 0.450729799 |  |  |
|  | 1.955560937 | 0.519789989 | 0.975180555 | 14 | C vs L ns  C vs H ns  L vs H ns |
|  | 0.637177462 | 1.01330058 | 3.008153774 |  |  |
|  | 1.442269593 | 2.113705148 | 1.554753614 |  |  |
|  | 0.459014087 | 2.288541113 | X |  |  |
|  | 0.562421567 | 0.282859258 | 0.275327696 |  |  |
|  | 0.69000575 | 0.778089462 | 1.30624727 | 21 | C vs L ns  C vs H ns  L vs H ns |
|  | 0.663463921 | 4.898801447 | X |  |  |
|  | 0.231693518 | 1.057596822 | 0.605020283 |  |  |
|  | 2.53410783 | 0.722974861 | 0.746653107 |  |  |
|  | 2.821904455 | 0.730746967 | 1.528718761 |  |  |
| *RF39_fa* | 1.40331862 | 0.553105314 | 0.196455013 | 0 | C vs L ns  C vs H ns  L vs H ns |
|  | 0.471064691 | 0.011312034 | 1.094751297 |  |  |
|  | 0.309755236 | 0.245511128 | 0.5047789 |  |  |
|  | 0.359476823 | 0.316076963 | 0.347320911 |  |  |
|  | X | 0.109462318 | 0.11955528 |  |  |
|  | 0.179360659 | 0.520444636 | 0.191370019 | 14 | C vs L ns  C vs H ns  L vs H ns |
|  | 0.192206424 | 0.307060782 | 0.686915304 |  |  |
|  | 0.167296229 | 0.184914572 | 0.268637443 |  |  |
|  | 0.1319006 | 0.379111861 | X |  |  |
|  | 0.227990985 | 0.072256882 | 0.798660492 |  |  |
|  | 0.357502979 | 0.439162634 | 0.417940877 | 21 | C vs L ns  C vs H ns  L vs H ns |
|  | 0.321340357 | 1.006331976 | X |  |  |
|  | 0.412198933 | 1.05405022 | 0.186278509 |  |  |
|  | 0.426599412 | 0.129444126 | 0.242193929 |  |  |
|  | 0.284173048 | 0.143283719 | 1.211437508 |  |  |


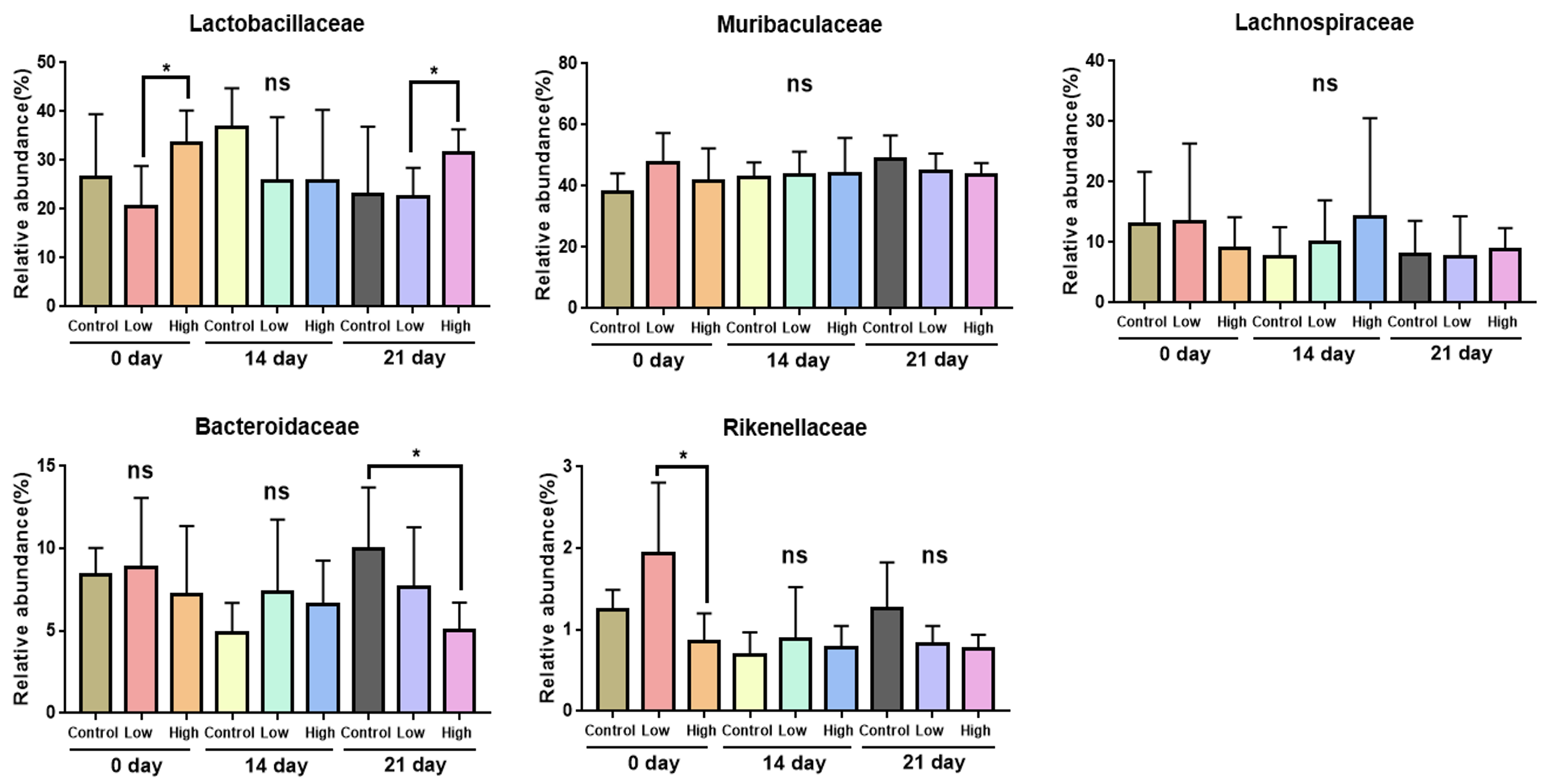


**Figure S1.** Relative abundance of gut microbiota at the main five family in green banana-gavaged mice on day 0, 14, 21. Unpaired t-tests (two-tailed) were used to analyze variations between the two groups. (*p< 0.05, ns means no significant)


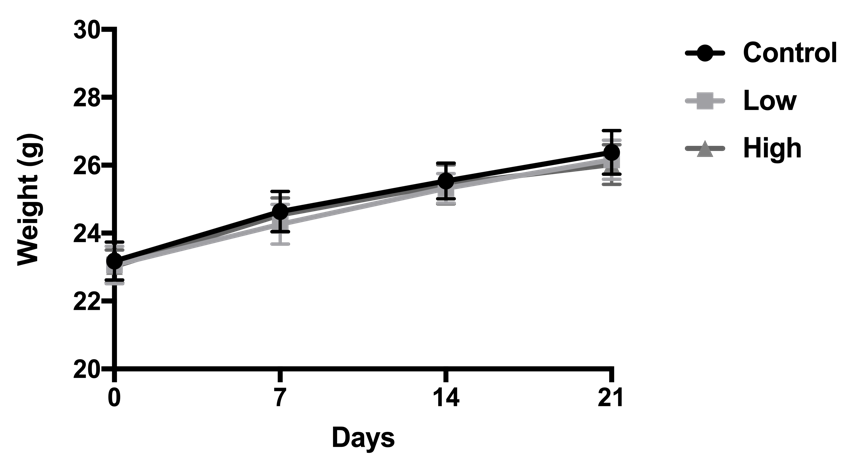


**Figure S2.** Comparison of the body weight of the mice in three groups by the time. Unpaired t-tests (two-tailed) were used to analyze variations between the two groups. There was no significant difference.
